# Point-of-Care Detection of Carcinoembryonic Antigen (CEA) Using a Smartphone-Based, Label-Free Electrochemical Immunosensor with Multilayer CuONPs/CNTs/GO on a Disposable Screen-Printed Electrode

**DOI:** 10.3390/bios14120600

**Published:** 2024-12-07

**Authors:** Supada Khonyoung, Praphatsorn Mangkronkaew, Puttaporn Klayprasert, Chanida Puangpila, Muthukumaran Palanisami, Mani Arivazhagan, Jaroon Jakmunee

**Affiliations:** 1 Department of Chemistry, Faculty of Science and Technology, Thammasat University, Pathum Thani 12120, Thailand; skhonyoung@gmail.com; 2 Research Laboratory for Analytical Instrument and Electrochemistry Innovation, Department of Chemistry, Faculty of Science, Chiang Mai University, Chiang Mai 50200, Thailandbeautiful_blue_sky2@hotmail.com (P.K.); chanida.pu@cmu.ac.th (C.P.);; 3 Research Laboratory on Advanced Materials for Sensor and Biosensor Innovation, Materials Science Research Center, and Center of Excellence for Innovation in Chemistry, Faculty of Science, Chiang Mai University, Chiang Mai 50200, Thailand

**Keywords:** smartphone, immunosensor, carcinoembryonic antigen, label-free detection, layer-by-layer assembly, lung cancer

## Abstract

In order to identify carcinoembryonic antigen (CEA) in serum samples, an innovative smartphone-based, label-free electrochemical immunosensor was created without the need for additional labels or markers. This technology presents a viable method for on-site cancer diagnostics. The novel smartphone-integrated, label-free immunosensing platform was constructed by nanostructured materials that utilize the layer-by-layer (LBL) assembly technique, allowing for meticulous control over the interface. Detection relies on direct interactions without extra tagging agents, where ordered graphene oxide (GO), carbon nanotubes (CNTs), and copper oxide nanoparticles (CuONPs) were sequentially deposited onto a screen-printed carbon electrode (SPCE), designated as CuONPs/CNTs/GO/SPCE. This significantly amplifies the electrochemical signal, allowing for the detection of low concentrations of target molecules of CEA. The LBL approach enables the precise construction of multi-layered structures on the sensor surface, enhancing their activity and optimizing the electrochemical performance for CEA detection. These nanostructured materials serve as efficient carriers to significantly increase the surface area, conductivity, and structural support for antibody loading, thus improving the sensitivity of detection. The detection of carcinoembryonic antigen (CEA) in this electrochemical immunosensing transducer is based on a decrease in the current response of the [Fe(CN)_6_]^3−/4−^ redox probes, which occurs in proportion to the amount of the immunocomplex formed on the sensor surface. Under the optimized conditions, the immunosensor exhibited good detection of CEA with a linear range of 0.1–5.0 ng mL^−1^ and a low detection limit of 0.08 ng mL^−1^. This label-free detection approach, based on signal suppression due to immunocomplex formation, is highly sensitive and efficient for measuring CEA levels in serum samples, with higher recovery ranges of 101% to 112%, enabling early cancer diagnosis. The immunosensor was successfully applied to determine CEA in serum samples. This immunosensor has several advantages, including simple fabrication, portability, rapid analysis, high selectivity and sensitivity, and good reproducibility with long-term stability over 21 days. Therefore, it has the potential for point-of-care diagnosis of lung cancer.

## 1. Introduction

Carcinoembryonic antigen (CEA) is an important tumor biomarker that is typically overexpressed in patients with various cancers, such as colorectal, lung, breast, and pancreatic cancers. These patients usually exhibit overexpression of this glycoprotein in their blood and tissues [1,2,3]. As a result, the early detection of CEA can significantly contribute to early cancer diagnosis, monitoring, and treatment outcomes. The most prevalent form of cancer and the primary global cause of cancer-related mortality is lung cancer [4,5,6,7]. Although smoking is one of the major risk factors for lung cancer, non-smokers are also at risk due to other factors, such as exposure to radon, asbestos, or air pollution, as well as genetic predispositions [8,9,10]. In addition, environmental factors, including atmospheric hazardous changes, family history of lung cancer, and occupational exposures, have been recognized as possible risk variables for lung tumorigenesis. Air pollution, particularly haze, has also been linked to an increased risk of lung cancer [11,12,13]. The Northern region of Thailand has been experiencing severe haze for the past few years, which has had a significant impact on public health. This situation has caused a rise in the number of patients with respiratory diseases and an increased risk of lung cancer [14,15,16]. Early detection and treatment are critical for improving survival rates, but lung cancer is often diagnosed at later stages when symptoms become apparent, making it more difficult to treat effectively. Traditional detection methods often require labeling agents such as fluorescent or radioactive tags, which add complexity, cost, and time to the process [17,18,19]. A label-free approach simplifies the detection by directly measuring the electrochemical signal changes caused by antigen-antibody binding. Since CEA is highly expressed in cancer patients, the ability to detect its presence at low concentrations is critical for early screening and diagnosis [20,21,22]. Efforts to integrate routine screening for high-risk populations, combined with advanced biomarker studies, will be key to reducing lung cancer mortality globally, which is the leading cause of cancer-related mortality worldwide. This can lead to earlier intervention, improving patient outcomes. This type of sensor has strong potential for clinical settings in cancer screening, making it a valuable tool in the fight against cancer [23,24,25].

The improvement of patient survival rates is contingent upon early detection of cancer. Given the high mortality rate associated with advanced lung cancer, promoting widespread access to screening and raising awareness about early detection is key to improving long-term survival rates [24,26]. In this context, sensitive and specific methods are needed for early cancer diagnosis. The identification of tumor markers in blood samples is an effective method for early cancer diagnosis [27]. Carcinoembryonic antigen (CEA) is a well-known glycoprotein utilized as a biomarker for various cancers, including lung cancer. CEA is present at low levels in healthy individuals but can be elevated in patients with certain malignancies, particularly in adenocarcinomas [28]. For lung cancer, CEA levels are often measured to help monitor disease progression, recurrence, and treatment efficacy, especially in non-small cell lung cancer (NSCLC) [13]. The average CEA concentration in a healthy person is 2.5–5 ng mL^−1^, while serum CEA levels above 5 ng mL^−1^ indicate a risk of cancer [13,29]. The conventional method for CEA detection is enzyme-linked immunosorbent assay (ELISA), but it has limitations, such as the need for expensive and large equipment, professional operators, complex operation, and slow analysis, which make it unsuitable for early diagnosis and prognostic monitoring. Thus, a simple, accurate, rapid, and cost-effective method for CEA analysis is required [30,31]. Electrochemical immunosensors represent a cutting-edge approach in modern diagnostics, offering the promise of early detection and personalized medicine through enhanced biomarker monitoring [32]. Label-free immunosensing platforms are revolutionizing diagnostics by enabling rapid, sensitive, and highly specific detection of biomarkers critical for early disease diagnosis and management [33]. Their integration into healthcare supports advancements in personalized medicine, as these devices provide real-time, quantitative data on disease-specific biomolecules [34]. Electrochemical immunosensors are bridging the gap between laboratory diagnostics and patient-centered care, offering unparalleled opportunities for non-invasive, fast, and accurate monitoring. Their potential to transform healthcare aligns with the goals of precision medicine and global health equity.

Indeed, from a point-of-care (POC) perspective, electrochemical immunosensors are a promising alternative to conventional diagnostic methods [35]. They offer significant advantages, particularly in the early detection of diseases like cancer, where timely diagnosis can dramatically improve outcomes. For diseases like lung cancer, where early detection is critical, electrochemical immunosensors can offer a faster and more accessible route to diagnosis at the point of care [36]. Their development is key to advancing personalized medicine, especially in resource-limited settings where conventional laboratory infrastructure may not be available. For instance, electrochemical immunosensors offer several advantages to overcome the drawbacks of conventional methods, such as portable sensors or analytical devices, including electrochemical sensors, microfluidic devices, or paper-based sensors. These types of devices are typically valued for their affordability, disposability, simplicity, and ease of use [37]. Interestingly, label-free immunosensors can directly detect changes in the signal of the antigen–antibody complex using redox probes, which greatly simplifies sensor fabrication and operation and does not require a secondary labeled antibody [25,31]. However, the performance of an accurate and sensitive immunosensor can be improved by using several nanomaterials for the construction of sensing interfaces. The utilization of nanomaterials, such as metal nanoparticles, carbon materials, magnetic materials, and polymeric materials, provides a high surface-to-volume ratio, enhancing the intrinsic properties of ionic and electrical conductivity, excellent biocompatibility, and high electron transportation ability [38]. The synergy between nanostructured materials and immunosensors holds the potential to revolutionize biosensing technologies in emerging biomarkers.

Graphene oxide (GO) is a two-dimensional (2D) monolayered carbon-based material with unique properties. It is composed of an exclusive layer of hexagonally arranged carbon atoms, much like graphene, but with oxygen-containing functional groups comprising carboxyl, epoxy, and hydroxyl groups, which are linked to its basal plane and edges and interact with other nanostructured materials such as metal oxides, hydroxides, and sulfides [39,40]. These oxygen functional groups make GO hydrophilic and give it the ability to form stable colloidal dispersions in water and other solvents. The presence of the π-conjugated system (delocalized π-electrons across the carbon atoms) gives GO its electrical conductivity (although reduced compared to pure graphene), and its large surface area makes it an excellent candidate [8]. The combination of these properties, such as its 2D structure, high surface area, and functional groups, allows GO to interact with various molecules and ions, making it a highly versatile electrode material. Its ability to load metal nanomaterials enhances the uniformity and dispersion of the metal nanomaterials, improving fast electron transportation and ionic conductivity due to the tiny-sized transition metal-based oxides, hydroxides, and sulfides [41,42,43]. The earth-abundant, first-row transition metal of non-precious copper oxide nanoparticles (CuONPs) is particularly interesting among other metal oxide nanoparticles due to their low cost compared to silver nanoparticles (AgNPs) and gold nanoparticles (AuNPs). Copper oxide nanoparticles (CuONPs) serve as the active sensing material due to their catalytic properties, making them appropriate for the sensing of carcinoembryonic antigen (CEA) via electrochemical reactions [24,44]. Carbon nanotubes (CNTs) have also been widely incorporated into the fabrication of electrochemical immunosensors due to their unique properties, including a large surface area-to-volume ratio, low background noise, high chemical stability, structural support, and excellent electrical conductivity. By enhancing the encapsulation of biological identification elements, expanding the active surface areas of electrodes, and enhancing electron transportation, carbon nanotubes (CNTs) can improve electrochemical sensors’ sensitivity and lower the threshold for their identification limit. In addition, CNTs are especially helpful in enhancing the functionality of electrochemical sensors [39,44].

Herein, we report a novel label-free electrochemical immunosensor for the sensitive and stable detection of CEA utilizing CuONPs/CNTs/GO-modified SPCE and a smartphone-based platform. We altered single-use, disposable screen-printed electrodes layer-by-layer with GO to improve the intrinsic properties, CNTs for structural support and stability, and CuONPs, and evaluated the analytical capabilities of the modified electrode for CEA detection using a redox probe-immunosensing strategy based on a label-free immunosensor. The nanomaterials in the CuONPs/CNTs/GO/SPCE sensor platform served as efficient carriers, significantly increasing the surface area for antibody loading. This is crucial for improving the sensitivity of detection, particularly in immunosensors. This label-free detection approach, based on signal suppression due to immunocomplex formation, is highly sensitive and efficient for measuring CEA levels in serum samples, enabling early cancer diagnosis. Synergistically, GO and CNTs offer unique surface chemistry and mechanical properties. The incorporation of GO and CNTs helps disperse the CuO nanoparticles more evenly, preventing aggregation and ensuring that the material retains its high surface area and reactivity [45]. Together, these materials form a highly conductive, robust platform with enhanced electrocatalytic performances and a large surface area, as well as improving the sensitivity of the sensor for CEA detection. The use of a screen-printed carbon electrode (SPCE) offers a low-cost, disposable, and flexible base, making it ideal for integrating with portable devices such as smartphones. This assembly increases the sensor’s high-precision ability to identify lower levels of CEA in serum samples, which raises the possibility that it will be useful for early cancer diagnosis. This innovative sensor platform’s ability to accurately detect lower levels of CEA in serum samples positions it as a cutting-edge instrument for early-stage cancer diagnostics, offering the potential for timely and effective cancer treatment.

## 2. Experiment

### 2.1. Chemicals and Materials

All the chemicals used in this study were of analytical reagent grade. This indicates that the chemicals meet high purity standards, ensuring minimal impurities. Mesoporous carbon (MC), copper oxide nanoparticles (CuONPs), silver nanosphere (AgNP), anti-carcinoembryonic antibody (anti-CEA), carcinoembryonic antigen (CEA), 1-ethyl-3-(3-dimethylaminopropyl) carbodiimide (EDC), Bovine Serum Albumin (BSA), and human serum samples used in this study were procured from Sigma-Aldrich (St. Louis, MO, USA). EDC, commonly used as a coupling agent in bioconjugation reactions, was procured from Spectrum Chemical Manufacturing Group, while BSA, often used as a stabilizing agent or blocking protein in biochemical assays, was procured from Merck (Boston, MA, USA). Human serum samples, crucial for testing the practical applicability of sensors or assays in a biological context, were obtained from Sigma-Aldrich (St. Louis, MO, USA). Graphene oxide (GO) and Carbon nanotubes (CNTs) were obtained from the Nanomaterial Research Unit at Chiang Mai University, Thailand. Potassium ferricyanide (K_3_Fe(CN)_6_) and potassium ferrocyanide (K_4_Fe(CN)_6_) were purchased from Sigma-Aldrich (Beijing, China). These compounds are often used in electrochemical experiments as redox probes due to their well-known reversible redox properties. N-hydroxysuccinimide (NHS), ordered from Sigma-Aldrich (Tokyo, Japan), is frequently used in biochemical conjugation reactions, often alongside EDC, to activate carboxyl groups for covalent bonding with amine groups, forming stable amide linkages. This reagent is essential for immobilizing biomolecules, such as antibodies, onto the SPCE. The 10 mM phosphate buffer saline solution (PBS, pH 7.4) was prepared by dissolving 0.25 g KH_2_PO_4_ (Sigma-Aldrich, Tokyo, Japan), 3.58 g Na_2_HPO_4_·2H_2_O (Sigma-Aldrich, Beijing, China), 0.20 g KCl (Ajax Finechem, Wollongong, Australia), and 8.0 g NaCl (Loba Chemie, Mumbai, India) in 800 mL DI water. The pH of the solution was adjusted using 0.10 M HCl, and DI water was added to a final volume of 1000 mL.

### 2.2. Apparatus and Methods

**Scheme 1:**
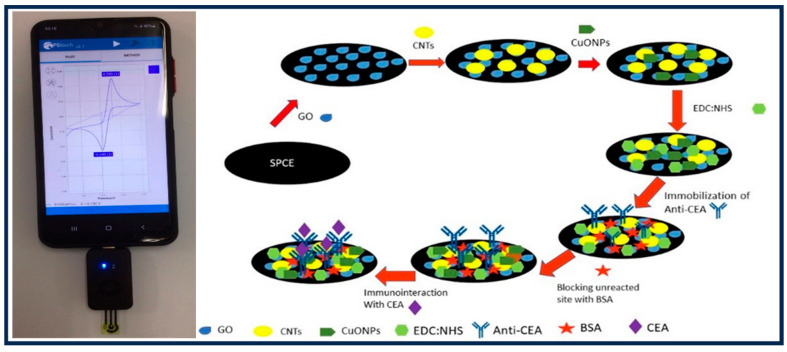
CEA detection using Sensit smartphone-based potentiostat and the fabrication process of the electrochemical immunosensor.

The performance of electrochemical sensors was evaluated using electrochemical measurements. Electrochemical impedance spectroscopy (EIS) provides insights into the interfacial properties of the sensor, including the charge transfer resistance, double-layer capacitance, and other impedance components. Cyclic voltammetry (CV) was utilized to analyze the redox behavior and kinetics of the electrochemical reactions on the modified SPCE’s exterior surface. All the electrochemical investigations were conducted using Sensit/Smart and PlamSens4 potentiostats (PalmSens, Houten, The Netherlands). Home-made screen-printed carbon electrodes (SPCEs) with a 3 mm-diameter working electrode were prepared following the method described by Reanpang et al. [46]. The SPCEs were treated with a plasma cleaner (PDC-32 G, Harrick, NY, USA) under the optimum condition at 0.4 torrs for 60 s to explore more active electrode sites and enhance the hydrophilic properties of the electrode surface. All electrochemical experiments were conducted using a redox couple of [Fe(CN)_6_]^3−/4−^ solution in a three-electrode electrochemical cell. The use of the ferri/ferrocyanide redox couple is common in electrochemical studies due to its well-defined and reversible redox reactions, which provide a reliable system for evaluating electrode performance. This setup allows precise control and measurement of electrochemical processes, facilitating the assessment of the sensor’s characteristics, such as electron transfer kinetics and surface interactions. The surface morphologies of the modified electrodes were investigated by a scanning electron microscope (JEOL JSM-IT300, Tokyo, Japan). To measure the oxidation current response of the immunosensor, an SPCE affixed to the Sensit/Smart small potentiostat device was used, with a 100 μL drop of 10 mM [Fe(CN)_6_]^3−/4−^ (in 10 mM, pH 7.4 PBS buffer). The experiment was carried out on a Samsung Galaxy A03s (Samsung, Seoul, Korea) smartphone, as shown in Scheme 1. SEM is an effective technique for closely examining the morphology and surface structure of nanomaterials. A scanning electron microscope (JEOL JSM-7100F, Tokyo, Japan) was used to characterize the morphological traits of the nanomaterials on the modified SPCE.

### 2.3. Fabrication of Home-Made, Screen-Printed Carbon Electrode (SPCE)

**Figure 1:**
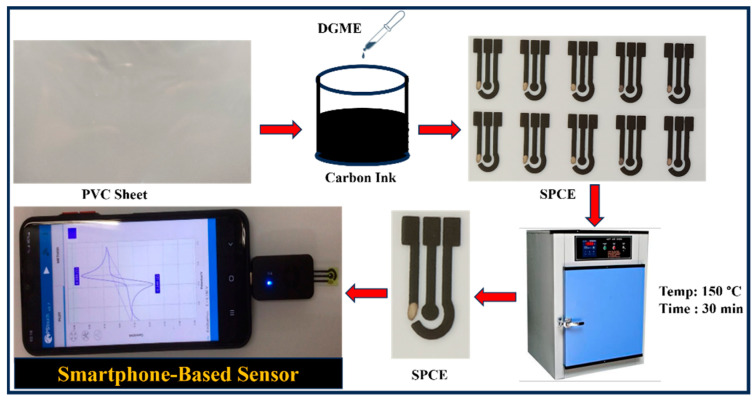
Schematic representation of the preparation for home-made SPCE for the sensing of CEA.

In the sensor fabrication process, Acheson Electrodag PR-406 highly conductive carbon ink (Henkel, Rocky Hill, CT, USA) was utilized to create the self-made fabrication process of screen-printed carbon electrodes (SPCEs) under laboratory conditions. This ink is commonly used due to its excellent electrical conductivity, adhesion, and ease of processing. This home-made fabrication process using Acheson Electrodag PR-406 carbon ink provides a cost-effective and scalable way to produce high-performance SPCEs for advanced electrochemical sensing platforms. A PVC sheet was employed as the base substrate for various electrochemical applications. The fabrication process (Figure 1) begins by diluting the highly conducting carbon ink with diethylene glycol monobutyl ether (DGME) (MERCK, Taufkirchen, Germany), which acts as the solvent to achieve the semi-solid paste with proper consistency of the substance. Initially, the prepared carbon ink was uniformly drop-coated on the PVC substrate and incubated at 150 °C for 30 min to fabricate the SPCE [47]. This step ensures proper adhesion and curing of the ink on the PVC substrate, resulting in durable and flexible electrodes. The procedure was repeated to strengthen the film attachment between the carbon particles and the substrate’s surface, producing a sheet with good conductivity. Plasma treatment was applied to enhance the surface characteristics of the SPCE, including wettability, adhesion, and surface energy. The duration and specific conditions of the plasma treatment can significantly impact the outcome of the electrocatalytic activity of the electrodes towards analytes.

### 2.4. Preparation of Multilayer Nanomaterials Sensors Based on GO

**Figure 2:**
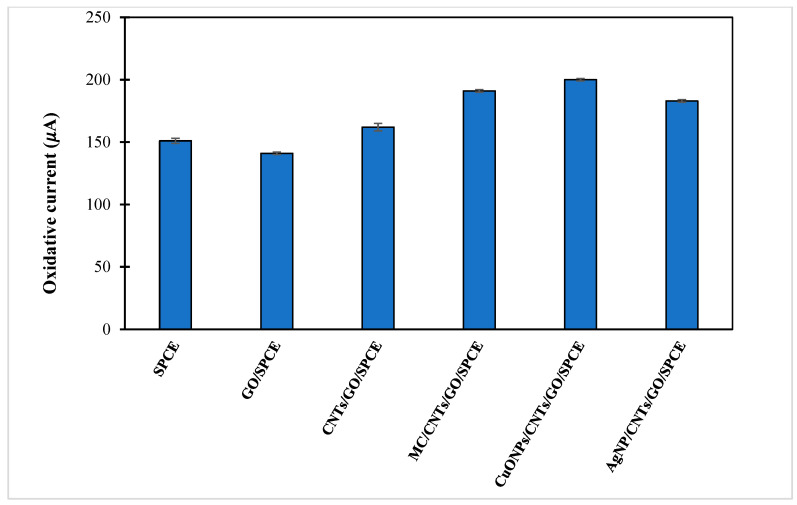
Current responses of bare and surface-modified SPCEs with various nanostructured materials in CV measurement with 10 mM [Fe (CN)_6_]^3−/4−^.

To optimize the analysis conditions, modified electrodes, including GO/SPCE, CNTs/GO/SPCE, MC/CNTs/GO/SPCE, CuONPs/CNTs/GO/SPCE, and AgNP/CNTs/GO/SPCE, were constructed by a sandwich-type layer-by-layer assembly. For each electrode, 10.0 µL of a nanomaterial suspension containing a disproportionate amount of 0.02 mg mL^−1^ was dropped onto the exterior surface of a plasma-activated SPCE and dried at room temperature. The oxidation current of 10 mM [Fe (CN)_6_]^3−/4−^ was measured using CV detection for each modified SPCE. The nanomaterials that provided the highest sensitivity were selected for further optimization. This is a common method for evaluating the electrochemical properties and performance of electrode exterior surface modifications, particularly in determining fast electron transfer kinetics and conductivity improvements due to nanomaterials. Graphene oxide (GO), known for its high electrical conductivity, is anticipated to improve the ratio of electron communication and enhance the intrinsic properties. CNTs provide structural stability, and CuONPs offer higher biocompatibility as well as overall performance. The modified electrode producing the highest oxidation current indicates better electrocatalytic activity and/or an enhanced surface area for electron transportation, warranting further optimization. These findings are displayed in Figure 2.

### 2.5. Fabrication of Multilayer Immunosensor for CEA Detection

The as-prepared CuONPs/CNTs/GO/SPCE was surface-activated by treating it with 10.0 μL of EDC (1-ethyl-3-[3-dimethylaminopropyl] carbodiimide hydrochloride) and NHS (N-hydroxysuccinimide) twice, for 30 min each time, and then dried at room temperature. This process plays a crucial role in facilitating the reaction between the carboxylic acid groups of graphene oxide (GO) and the amine groups of the anti-CEA antibody molecules. This process, often referred to as covalent immobilization, enhances the stability and specificity of antibody attachment, which is vital for the overall performance of the sensor in detecting carcinoembryonic antigen (CEA) [48]. Subsequently, the altered electrode was subjected to a 60-min incubation period with 10.0 μL of anti-CEA (1 μg mL^−1^). The electrode was thoroughly cleaned with deionized (DI) water to remove unbound antibodies and then dried at 4 °C. This cleaning process is essential in immunosensing platforms as it ensures that only specifically bound antibodies remain, enhancing the specificity and sensitivity of the sensors while minimizing background noise and interferences. In order to prevent indiscriminate binding sites on the electrode surface, the anti-CEA/CuONPs/CNTs/GO/-modified SPCE was incubated with 10.0 μL of 1 μg mL^−1^ bovine serum albumin (BSA) twice for 2 h each time and repeated DI water washing. The prepared electrodes were then incubated with 10.0 μL of CEA solutions at different concentrations (0.1–10.0 ng mL^−1^) for 30 min and washed with DI water several times. The fabrication steps are depicted in Scheme 1**.**

## 3. Results and Discussion

### 3.1. Electrochemical Properties of Multilayer Nanomaterial Sensors

Surface modifications with nanomaterials significantly enhance the orientation and accessibility of the immobilized biomolecules and maximize their binding efficiency to target analytes. These modifications exhibit both the adsorption capacity of bioactive substances and the electron transport ability [49,50]. In this study, various carbon nanomaterial-modified immunosensors for CEA detection were constructed, and the oxidative current of different modified electrodes was evaluated using 10 mM [Fe(CN)_6_]^3−/4−^, as shown in Figure 1. The electrical current for GO/SPCE was slightly lower than that of the bare SPCE electrode due to its poor conductivity. To overcome this issue, CNTs were used to modify the GO/SPCE electrode, leading to a rise in oxidative current due to the highly conductive nature and surface stability of CNTs. Furthermore, in order to enhance the qualities of the CNTs/GO/SPCE, nanomaterials such as MC, CuONPs, and AgNPs were combined with the CNTs/GO/SPCE, resulting in a highly conductive surface with a large surface area that significantly enhances their conductivity and electron transfer property.

The results obtained were consistent with the aforementioned hypothesis. The oxidative currents of all the prepared electrodes were higher than those of CNTs/GO/SPCE. Therefore, CuONPs/CNTs/GO/SPCE provided the highest oxidation (*i*_pa_) current, indicating its superior ability to enhance the electrochemical performance with the standard [Fe(CN)_6_]^3−/4−^ probe.

### 3.2. Morphological Characteristics

**Figure 3:**
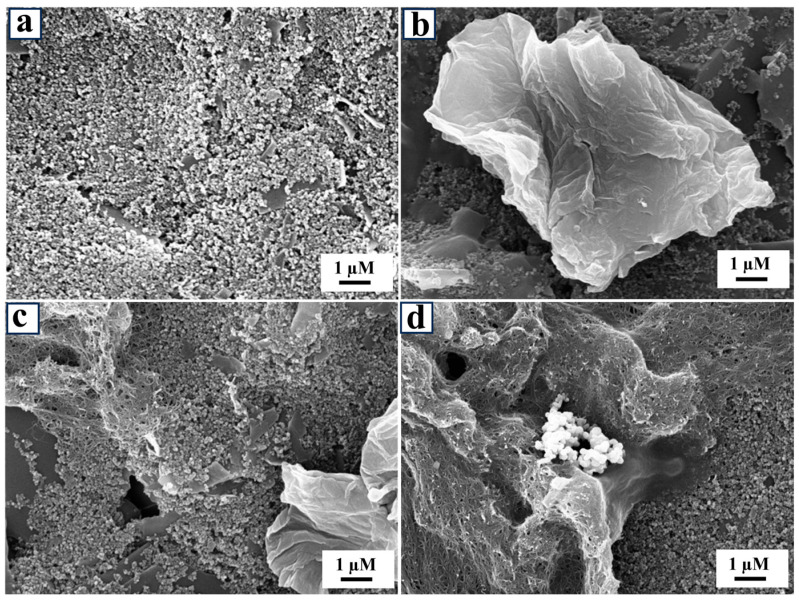
SEM images of (**a**) unmodified SPCE, (**b**) GO nanosheets modified with SPCE, (**c**) CNTs/GO/SPCE, and (**d**) CuONPs/CNTs/GO/SPCE.

**Figure 4:**
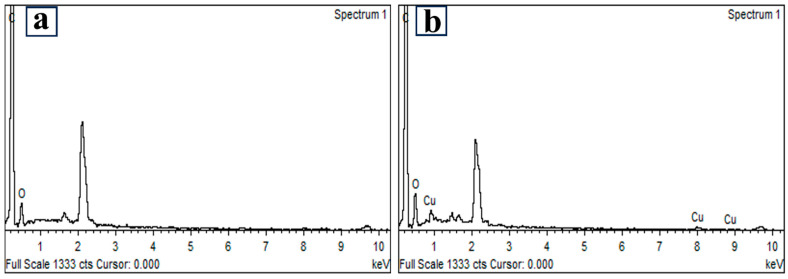
EDS spectra of (**a**) CNTs/GO/SPCE and (**b**) CuONPs/CNTs/GO/SPCE.

The modified electrodes’ surface morphologies were investigated using Field Emission Scanning Electron Microscopy (FE-SEM). This technique enables high-resolution imaging, allowing detailed examination of surface characteristics, including the drop-casting of nanostructures on the modified electrodes. SEM analysis was performed to assess the morphologies of the CuONPs/CNTs/GO/SPCE at every stage of the fabrication procedure, as shown in Figure 3. The GO material was found to have a wrinkled, sheet-like structure when coated onto the electrode surface, as depicted in Figure 3b. The CNTs exhibited a bundle of filaments, providing enhanced material support and improving the intrinsic properties of the electrodes, as shown in Figure 3c. In addition, numerous spherical CuONPs were incorporated into the modified electrodes, clearly shown in Figure 3d. Figure 4 displays the EDS spectra of CNTs/GO/SPCE and CuONPs/CNTs/GO/SPCE. Compared to Figure 4a, the characteristic elemental peaks corresponding to Cu were distinctly seen in Figure 4b, indicating the successful attachment of CuONPs to the electrode surface.

### 3.3. Electrochemical Behaviour of the Immunosensor

**Figure 5:**
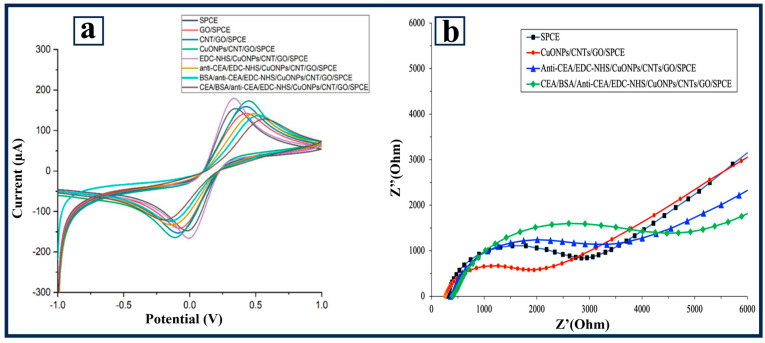
(**a**) Cyclic voltammograms and (**b**) EIS spectra of modified SPCEs in contact with 10 mM [Fe(CN)_6_]^3−/4−^.

CV and EIS investigations are essential tools for verifying the sequential modification steps on the immunosensor surface and ensuring the correct functionality of the electrode. CV helps assess the electrochemical behavior, while EIS probes the electrical properties and surface modifications of electrodes, as shown in Figure 5. CV assessments were carried out at every stage of the SPCE modification, as demonstrated in Figure 5a. It was noted that the redox current responses of [Fe(CN)_6_]^3−/4−^ significantly decreased after CEA binding with the prepared immunosensor due to the insulation properties of biomolecules present on the electrode surface after antibody and antigen binding [51]. The EIS Nyquist plot of the modified electrode was also investigated, providing insights into charge transfer resistance (R_ct_), double-layer capacitance (C_dl_), and the overall electrochemical behavior of the electrode surface. The results are summarized in Figure 5b. The impedance of the CuONPs/CNTs/GO/SPCE was lower than that of the bare SPCE, owing to the enhancement of the electroactive surface area on the outer surface electrode by the modified electrode materials. These findings provide a comprehensive understanding of the electrochemical characteristics of the altered electrodes. This holistic view reinforces the reliability and efficacy of the sensor design, making it more applicable for practical use in biomarker detection and diagnostics. The addition of an anti-CEA antibody on the exterior surface of the modified electrode increased the resistance, as expected. Finally, the magnitude of electron transfer resistance was reduced with the formation of the immunocomplex between the anti-CEA antibody and CEA antigen. The observed decrease in electron transfer resistance is due to immunocomplex formation on the electrode’s surfaces.

### 3.4. Optimization Steps to Fabrication of Nanostructured Materials

**Figure 6:**
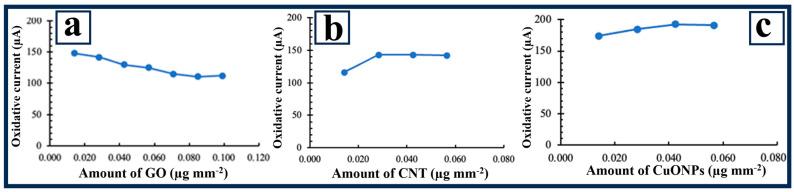
Effect of (**a**) amount of GO, (**b**) amount of CNTs, and (**c**) amount of CuONPs on the oxidative current.

In order to achieve a more intense electrochemical response, the amount of nanomaterials was optimized by drop-casting 5.0 μL of each 0.02 mg mL^−1^ nanomaterials onto the SPCE until the oxidative current became slightly constant, as shown in Figure 6. The resulting stable oxidative currents were observed at 0.071 μg GO mm^−2^, 0.028 μg CNTs mm^−2^, and 0.028 μg CuONPs mm^−2^. Therefore, these optimized conditions were selected for further experiments.

### 3.5. Analytical Performance

**Figure 7:**
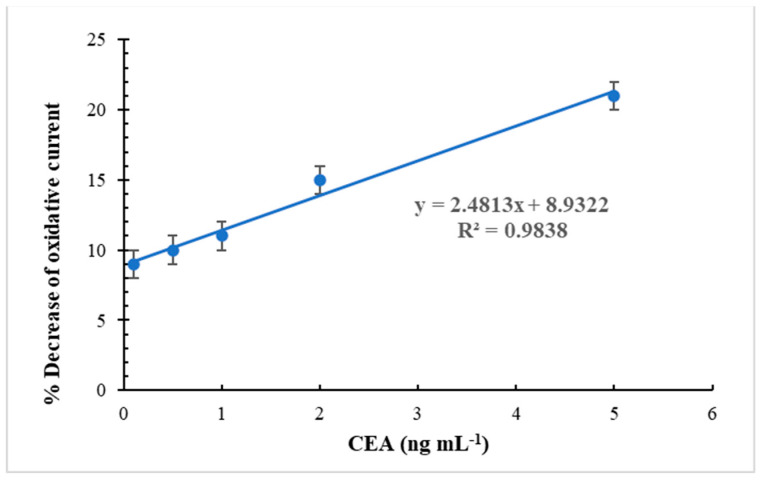
The plots show the percentage decrease in oxidative current versus CEA concentration.

The anti-CEA/CuONPs/CNTs/GO/SPCE immunosensing platform was investigated for the outcome detection of CEA under ideal conditions. The oxidative current response decreased gradually with raising the concentrations of CEA, and the peak current obtained from CV at about 0.5 V vs. Ag/AgCl reference electrode was utilized to calculate a percentage decrease in oxidative current relative to the peak current obtained prior to incubation with the CEA standard or sample. A calibration graph of the percentage decrease of oxidative current versus the concentration of CEA was constructed for concentration ranges of 0.1–5.0 ng mL^−1^. The regression equation was y = 2.4813x + 8.9322 (R^2^ = 0.9838) (Figure 7). The limit of detection (LOD) was 0.08 ng mL^−1^, based on a signal-to-noise ratio of 3 (S/N = 3). This LOS is lower than the warning CEA level of normal people (usually 5 ng mL^−1^) in serum. The performance of the developed CEA immunosensor was compared with previously reported sensors using [Fe(CN)_6_]^3−/4−^ as a redox probe, as presented in Table 1. The proposed immunosensor for carcinoembryonic antigen (CEA) detection offers significant advantages due to its ease of fabrication and portability. By integrating the sensor with a smartphone-based mini-potentiostat, the platform becomes more user-friendly and accessible, allowing for on-site and real-time detection without the need for bulky lab equipment. This combination of simplicity in construction and mobile device compatibility enhances its potential for point-of-care diagnostics.

### 3.6. Evaluation of the Selectivity, Reproducibility, and Stability of the Immunosensor

**Figure 8:**
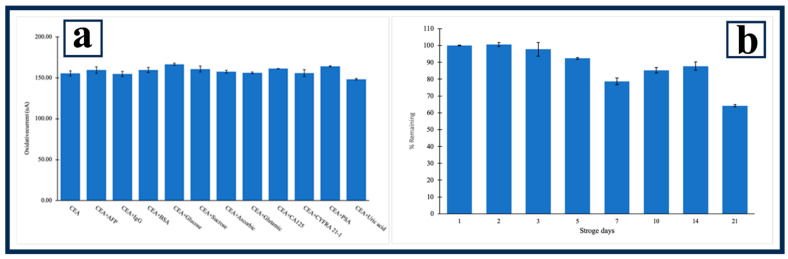
(**a**) Oxidative current of the interference substances (200 ng mL^−1^) mixed with CEA (2 ng mL^−1^) solution. (**b**) Stability of the sensor for different days (n = 3).

In this investigation, a high concentration (200 ng mL^−1^) of various substances was added in order to evaluate the selectivity, including cancer antigen 125 (CA125), alpha-fetoprotein (AFP), glucose, bovine serum albumin (BSA), immunoglobulin G (IgG), sucrose, ascorbic acid, glutamic acid, cytokeratin 19 fragment (CYFRA 21-1), prostate-specific antigen (PSA), and uric acid. The test was carried out by mixing each interfering substance with 2 ng mL^−1^ of CEA. Despite the concentration of interferences being 100 times higher than CEA, the oxidative current did not significantly change after mixing these interfering substances with CEA, as shown in Figure 8a. This result indicates that the proposed immunosensor is highly selective for CEA. The reproducibility of the immunosensor was investigated by preparing nine immunosensors for CEA detection at 1.0 and 5.0 ng mL^−1^. The relative standard deviation (RSD) was 5.3% and 4.8%, respectively, suggesting that the reproducibility of the immunosensor is acceptable. The immunosensor’s stability was investigated by measuring the current response of CEA (2.0 ng mL^−1^) after storing the biosensors at 4 °C from 1 to 21 days. The immunosensor’s current response retained 64% at 21 days, as shown in Figure 8b, compared with the results obtained from the freshly prepared sensor. Based on the previously mentioned results, it can be concluded that the suggested immunosensor’s specificity, repeatability, and stability were satisfactory. The satisfactory performance in these areas positions the anti-CEA/CuONPs/CNTs/GO/SPCE immunosensing platform as a promising tool for clinical diagnostics. Its potential for reliable and sensitive detection of CEA can significantly contribute to the early diagnosis and monitoring of cancer. Repeatability and reproducibility tests indicated consistent performance across multiple measurements and different conditions. Additionally, the immunosensor exhibited long-term stability, maintaining its electrochemical characteristics over time and specificity in distinguishing CEA from potential interferents.

### 3.7. Analysis of Real Samples

In order to track the analytical dependability and potential applications of the created immunosensing platform, experiments were conducted using serum samples. The analytical reliability of the proposed anti-CEA/CuONPs/CNTs/GO/SPCE immunosensor was thoroughly evaluated by determining the recoveries of various concentrations (0.1, 1.0 and 5.0 ng mL^−1^) of CEA standard solutions in human serum samples. Human serum was diluted 100-fold with 10 mM PBS buffer solution (pH 7.4) before being used in order to avoid interference from non-specific proteins in the serum. As previously reported, the CEA level for early diagnosis and prognosis of lung cancer is above 5.0 ng mL^−1^, so the as-prepared biosensor is suitable for detecting CEA in the diluted serum sample. The obtained recovery ranges from 101% to 112%, as shown in Table 2, indicating that the immunosensor is applicable for analyzing real human serum samples. The biosensor’s performance in clinical settings, specifically for the analysis of serum samples, demonstrates high accuracy and dependability, as its results closely match reference values with real samples.

## 4. Conclusions

In this study, a new label-free and sandwich-type electrochemical immunosensor was fabricated using GO, CNTs, and CuONPs multilayer modification for portable sensing of CEA in serum samples. The proposed immunosensor, which can be used with a smartphone, exhibited a low detection limit of 0.08 ng mL^−1^, high selectivity, excellent reproducibility, and acceptable stability over 21 days for CEA detection in human serum samples. A major highlight of this work is that the preparation of the immunosensor and its detection mechanisms do not require a secondary antibody for labeling. Furthermore, compared to conventional CEA detection methods like ELISA, the developed immunosensor is easy to operate, involves no tedious steps, and requires simple instrumentation. Therefore, the developed immunosensor is a promising alternative method to facilitate clinical analysis. While this biosensor can only detect one biomarker, the simplicity and convenience of the method, along with its high sensitivity and selectivity, make it valuable for screening analysis. In the future, if the sensor can simultaneously detect multiple biomarkers, it may aid in the more accurate diagnosis of cancer. Overall, the development of this immunosensor is a significant advancement in clinical analysis, providing a straightforward and convenient means of detecting CEA in human serum samples with high recoveries and RSD values ranging from 0.30 to 1.38%.

## Figures and Tables

**Table 1:** A comparative analysis of the analytical performance of the proposed immunosensor with other nanostructured-based electrochemical CEA immunosensing platforms utilizing [Fe(CN)_6_]^3−/4−^ as a redox probe.

Modification Electrode	Stability (Day)	Linear Range (ng mL^−1^)	LOD (ng mL^−1^)	Ref.
CS/CNTs/GNPs/GCE	30	0.1–2.0 2.0–200	0.04	[52]
AuPtNWs/THI/rGO/GCE	15	5 × 10^−5^–100	6 × 10^−6^	[53]
thGP-thCTS-AuNPs/GCE	30	0.3–8.0 8.0–100	0.03	[54]
AuNPs/PEDOT/GR/GCE	15	0.0004–40	0.0001	[55]
AuNPs/CNOs/SWCNTs/CS/GCE	28	10^−4^–400	0.0001	[56]
PtNPs@rGO@PS NSs/GCE	28	0.05–70	0.01	[57]
rGO/GCE	NR	0.1–5.0	0.05	[58]
CuONPs/CNTs/GO/SPCE	21	0.1–5.0	0.08	This work

rGO: reduced graphene oxide, AuPtNWs: Au-Pt nanowire, GCE: glassy carbon electrode, THI: thionine, thGP: thiol graphene, PEDOT: poly(3,4-ethylenedioxythiophene), thCTS: thiol chitosan, GR: graphene, CNOs: carbon nano-onions, GNPs: Gold nanoparticles, SWCNTs: single-walled carbon nanotubes, PS NSs: polystyrene nanospheres, CS: chitosan, NR: not reported.

**Table 2:** Determination of CEA in real samples.

No.	Add (ng·mL^−1^)	Found (ng·mL^−1^)	Recovery (%)	RSD (%)
1	0.1	0.14 ^a^	-	0.41
2	1.0	1.10	112	1.38
3	5.0	5.04	101	0.30

^a^ This value is lower than the LOQ (0.26 ng mL^−1^) of the proposed immunosensor, so it was reported as the BLQ (Below the Limit of Quantification).

## Data Availability

All data are provided in the manuscript.
